# Deep Learning and Machine Vision Approaches for Posture Detection of Individual Pigs

**DOI:** 10.3390/s19173738

**Published:** 2019-08-29

**Authors:** Abozar Nasirahmadi, Barbara Sturm, Sandra Edwards, Knut-Håkan Jeppsson, Anne-Charlotte Olsson, Simone Müller, Oliver Hensel

**Affiliations:** 1 Department of Agricultural and Biosystems Engineering, University of Kassel, 37213 Witzenhausen, Germany; 2 School of Natural and Environmental Sciences, Newcastle University, Newcastle upon Tyne NE1 7RU, UK; 3 Department of Biosystems and Technology, Swedish University of Agricultural Sciences, 23053 Alnarp, Sweden; 4 Department Animal Husbandry, Thuringian State Institute for Agriculture and Rural Development, 07743 Jena, Germany

**Keywords:** convolutional neural networks, livestock, lying posture, standing posture

## Abstract

Posture detection targeted towards providing assessments for the monitoring of health and welfare of pigs has been of great interest to researchers from different disciplines. Existing studies applying machine vision techniques are mostly based on methods using three-dimensional imaging systems, or two-dimensional systems with the limitation of monitoring under controlled conditions. Thus, the main goal of this study was to determine whether a two-dimensional imaging system, along with deep learning approaches, could be utilized to detect the standing and lying (belly and side) postures of pigs under commercial farm conditions. Three deep learning-based detector methods, including faster regions with convolutional neural network features (Faster R-CNN), single shot multibox detector (SSD) and region-based fully convolutional network (R-FCN), combined with Inception V2, Residual Network (ResNet) and Inception ResNet V2 feature extractions of RGB images were proposed. Data from different commercial farms were used for training and validation of the proposed models. The experimental results demonstrated that the R-FCN ResNet101 method was able to detect lying and standing postures with higher average precision (AP) of 0.93, 0.95 and 0.92 for standing, lying on side and lying on belly postures, respectively and mean average precision (mAP) of more than 0.93.

## 1. Introduction

Computer vision techniques, either three-dimensional (3D) or two-dimensional (2D), have been widely used in animal monitoring processes and play an essential role in assessment of animal behaviours. They offer benefits for the monitoring of farm animals due to the wide range of applications, cost and efficiency [1]. Examples of employing machine vision techniques in monitoring of animal health and behaviour have been reviewed [1,2,3]. In monitoring of pigs, interpretation of animal behaviours, particularly those relating to health and welfare as well as barn climate conditions, is strongly related to their locomotion and posture patterns [3,4]. In order to determine lying and standing postures in pigs, 3D cameras have been widely used, due to their possibility of offering different colours in each pixel of an image based on the distance between the object and the depth sensor. One such effort was reported by [5], in which the monitoring of standing pigs was addressed by using the Kinect sensor. In this research, initially noise from depth images was removed by applying a spatiotemporal interpolation technique, then a background subtraction method was applied to detect standing pigs. Lao et al. [6] recognized lying, sitting and standing behaviours of sows based on 3D images. In another study, a Kinect sensor was also employed to localize standing pigs in depth images for automatic detection of aggressive events [7]. 2D images have also been used for locomotion and lying posture detection, which were mainly based on pixel movement [8] or features of ellipses fitted to the animals [9,10]. 

In most of the studies, due to different light sources and resolution (quality) of captured images, problems of a high level of noise and the generation of a great deal of data cause challenges for machine vision-based monitoring of livestock. It has been reported that machine learning techniques have the possibility to tackle these problems [11,12]. The most popular machine learning techniques employed for analysis of optical data for monitoring of pigs are included models (i.e., linear discriminant analysis (LDA), artificial neural networks (ANN), support vector machine (SVM)). However, in recent years, deep learning approaches, a fast-growing field of machine learning, have been used in image classification, object recognition, localization and object detection [13]. Deep learning is similar to ANN, but with deeper architecture and the ability for massive learning capabilities which leads to higher performance [14]. Deep learning has recently been applied in 2D and 3D pig-based machine vision studies. An example is the separation of touching pigs in 3D images using a low-cost Kinect camera, based on the convolutional neural network (CNN), with high accuracy of around 92% [15]. 

Deep learning has also been used for detection and recognition of pigs’ or sows’ behaviours in different imaging systems. A detection system based on the Kinect v2 sensor, the faster regions with convolutional neural network features (Faster R-CNN) technique in combination with region proposal network (RPN) and Zeiler and Fergus Net (ZFnet), were developed by [16] to identify different postures of sows (i.e., standing and sitting behaviours, and sternal, ventral and lateral recumbency). Yang et al. [17] used deep learning for automatic recognition of sows’ nursing behaviours in 2D images, with an accuracy of 97.6%, sensitivity of 90.9% and specificity of 99.2%. Faster R-CNN and ZFnet were also employed to recognize individual feeding behaviours of pigs by [18], where each pig in the barn was labelled with a letter. Their proposed method was able to recognise pigs’ feeding behaviours with an accuracy of 99.6% during the study. Segmentation of sow images from background in top view 2D images was also addressed by means of Fully Convolutional Network (FCN), built in Visual Geometry Group Network with 16 layers (VGG16), in another study by [19]. Their results showed the possibility of employing deep learning approaches for segmentation of the animal from different background conditions. 

Due to the fast growth of pig production around the world, the issue of real-time monitoring of the animals, particularly in large scale farms, becomes more crucial [1]. Monitoring of an individual pig’s posture during the whole lifetime in large scale farms is almost impossible for farmers or researchers, due to the labour- and time-intensive nature of the task. Utilizing state-of-the-art machine learning, along with machine vision techniques, for monitoring of groups of pigs in different farming conditions could offer the advantage of early problem detection, delivering better performance and lower costs. Thus, the main objective of this study was to develop a machine vision and a deep learning-based method to detect standing and lying postures of individual pigs in different farming conditions, including a variety of commercial farm systems. 

## 2. Material and Methods 

### 2.1. Housing and Data Collection

The data sets for this study were derived from three different commercial farms in Germany and Sweden. In Germany, two farms for weaning and fattening of commercial hybrids of Pietrain× (Large White × Landrace) were selected. A set of four pens in a room in each farm were chosen; these had fully slatted floors with central concrete panels and plastic panels on both sides (weaning farm), and fully slatted concrete floors (fattening farm). In a Swedish fattening farm, two rooms with two pens were selected, each having part-slatted concrete flooring with some litter on the solid concrete in the lying area. Pigs were of commercial Hampshire × (Landrace × Large White) breeding. 

**Figure 1:**
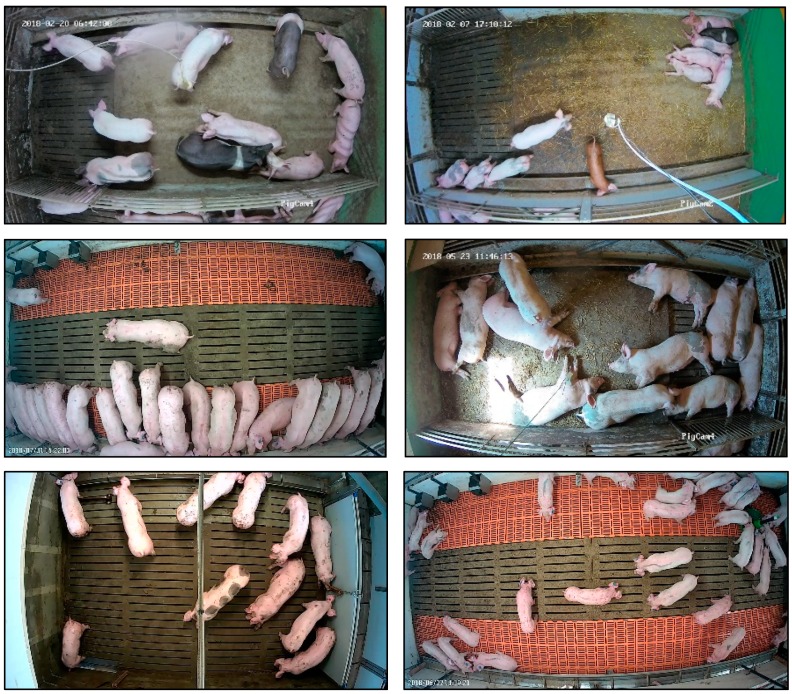
Example of images used for development of the detection algorithms in this study.

The images used in this study were recorded by top view cameras (VIVOTEK IB836BA-HF3, and Hikvision DS-2CD2142FWD-I). The cameras were connected via cables to servers and video images from the cameras were recorded simultaneously and stored on hard disks. Recording in different farming conditions allowed for capture of images with pigs of different skin colour and age, housed under various floor type and illumination conditions, which facilitated the development of a robust deep learning model. Examples of images used for development of the detection model are illustrated in Figure 1. 

### 2.2. Proposed Methodology

**Figure 2:**
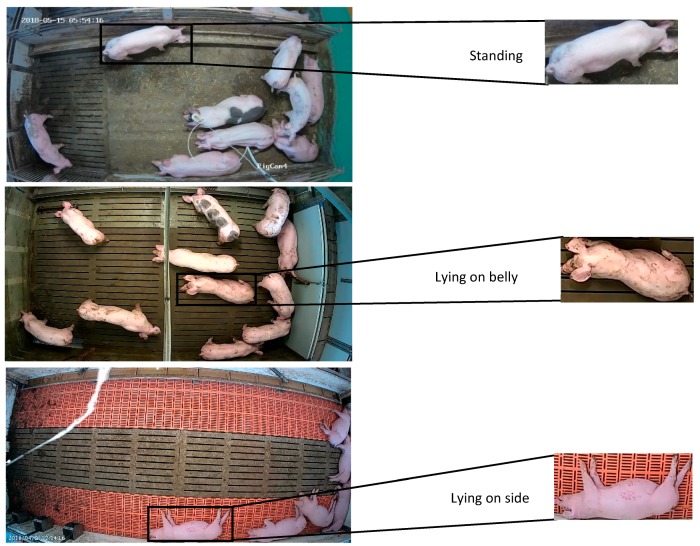
Examples of three posture classes used in this study in different farming conditions.

According to the findings of Nasirahmadi et al. [12], when in a side (lateral) lying posture pigs lie in a fully recumbent position with limbs extended, and when in a belly (sternal) lying posture the limbs are folded under the body. Due to the lack of available benchmark datasets for pigs’ lying and standing postures, three classes of standing, lying on belly and lying on side were defined in this work. Examples of these postures are represented in Figure 2. Most of the possible scenarios of individual pig standing and lying postures within the group have been considered. The proposed detection methods were developed and implemented in python 3.6 and OpenCV 3.4 using TensorFlow [20]. Experiments and the deep learning training were conducted on a Windows 10 with a NVIDIA GeForce GTX 1060 GPU with 6GB of memory. 

In order to establish the lying and standing postures, images from the three commercial farms over a period of one year were collected from surveillance videos. A total of 4900 images (4500 for training and validation, and 400 for testing) from this dataset, which incorporated various farm, animal age, and animal and background colour conditions, were selected to provide the training (80%: 3600) and validation (20%: 900) datasets. The images were selected at 1–2 week intervals from the beginning to the end of every batch, and different samples of images were collected randomly in one day of these selected weeks. Furthermore, another 400 images were taken randomly from the image data set as a testing set. The testing data were independent of the training and validation sets, and were used for an evaluation of the detection phase. All images were first resized to 1280 × 720 (width × height), then annotated using the graphical image annotation tool LabelImg, created by [21]. The annotations included the identification of standing and lying postures and were saved as XML files in the PASCAL VOC format. Since each image included different pigs (7–32 in number) based on farm conditions and time of recording, the total number of labelled postures was 52,785. The information on each posture class is summarized in Table 1. 

In this study, various combinations of deep learning-based models (i.e., Faster R-CNN (Inception V2, ResNet50, ResNet101 and Inception-ResNet V2), R-FCN (ResNet101) and SSD (Inception V2)) were developed with the aim of finding the best posture detection technique in 2D images. Two architectures of the ResNet model (i.e., ResNet50 and ResNet101) were applied. ResNet is a deep residual network introduced by [22] and has been widely used in object detection and localization tasks. Both ResNet50 and ResNet101 are based on repeating of four residual blocks. These feature extractors are made of three convolution layers of 1 × 1, 3 × 3 and 1 × 1. They also have an additional connection joining the input of the first 1 × 1 convolution layer to the output of the last convolution 1 × 1 layer. Additionally, residual learning in the ResNet model resolves the training by fusing filtered features with original features. ResNet50 and 101 are very deep networks and contain 50 and 101 layers. ResNet applies skip connections and batch normalization and provides short connection between layers [23]. Furthermore, ResNets allow direct signal propagation from the first to the end layer of the network without the issue of degradation. Inception V2 GoogLeNet [24] with layer-by-layer structure was also implemented as a feature extractor of the input pig posture images. The feature extractors which map input images to feature maps, characterize computational cost as well as performance of networks. Inception V2 GoogLeNet is based on the repetition of building blocks and extracts features concatenated as output of the module [24]. It is made of a pooling layer and different convolutional layers. The convolution layers are of different sizes of 1 × 1, 3 × 3, 5 × 5 and help to extract features from the same feature map on different scales and improve performance. Inception V2, by factorization of filters n×n to a combination of 1 × n and n × 1 convolutions, has led to better performance and reduction in representational bottlenecks. This has resulted in the widening of the filter banks instead of deepening, to remove the representational bottleneck without increase in computational cost and number of parameters [25]. Finally, Inception-ResNet V2, which was proposed in 2017 [26], was utilized as a feature extractor in this work. Inception-ResNet V2 is one of the state-of-the-art approaches in image classification and is based on combination of residual connections and Inception architectures. Inception-ResNet V2 is an Inception style network which uses residual connections instead of filter concatenation [26]. Further details on all parameters of the proposed feature extractors can be found in [22,24,26].

#### 2.2.1. Faster R-CNN 

**Figure 3:**
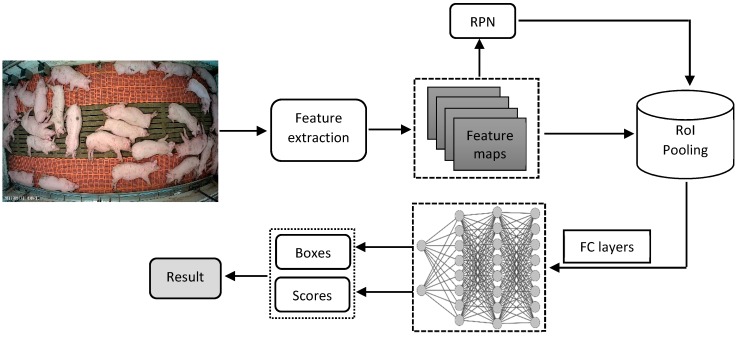
Schematic diagram of the faster regions with convolutional neural network (Faster R-CNN) used in this study.

The Faster R-CNN method [27] was used to monitor standing, side and belly lying postures of pigs. Figure 3 shows the architecture and different steps of the Faster R-CNN utilized in posture detection. Various feature extraction methods (Inception V2, ResNet50, ResNet101 and Inception-ResNet V2) were applied and the regional proposal network (RPN), which is a fully convolutional network for generating object proposals, was used to produce a proposed region for each image and generate feature maps in the final layer by predicting the classes [25]. The feature maps were fed into the region of interest (RoI) pooling to extract regions from the last feature maps. Each RoI (region of pigs) was then determined to a confidence score. Finally, the feature vector from the RoI was fed into several fully connected (FC) layers of a softmax classifier to obtain the final assurance scores and a regression bounding box to localize the coordinates of the detected objects. 

#### 2.2.2. R-FCN 

**Figure 4:**
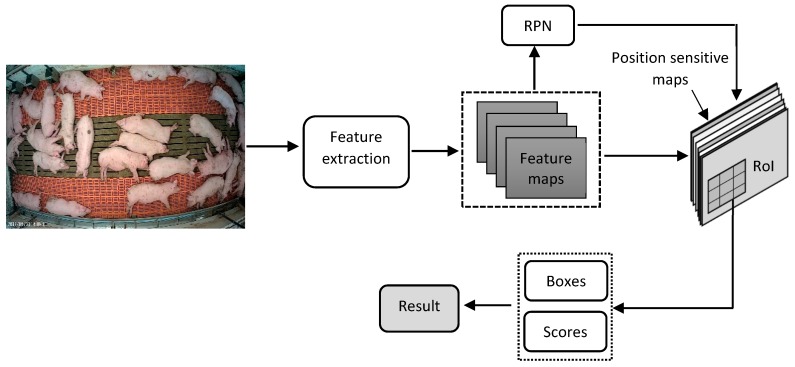
Schematic diagram of the region-based fully convolutional network (R-FCN) used in this study.

R-FCN [28] is an improvement of the Faster R-CNN and is proposed as an object detection technique using CNNs. The structure of the proposed R-FCN applied in this study is shown in Figure 4. The R-FCN consists of a feature extractor (ResNet101 in this study) and several FC layers behind an RoI pooling layer. The RoI pooling layer uses position sensitive maps to address the issue of translation invariance [29]. In the R-FCN, the computation is shared across the whole image by creating a deeper FCN without increasing the speed overhead. Region proposal and classification are done by use of RPN, followed by the position sensitive maps and RoI pooling, respectively. For training of the R-FCN model in this study, the same hyperparameters as the faster R-CNN were used. 

#### 2.2.3. SSD

**Figure 5:**
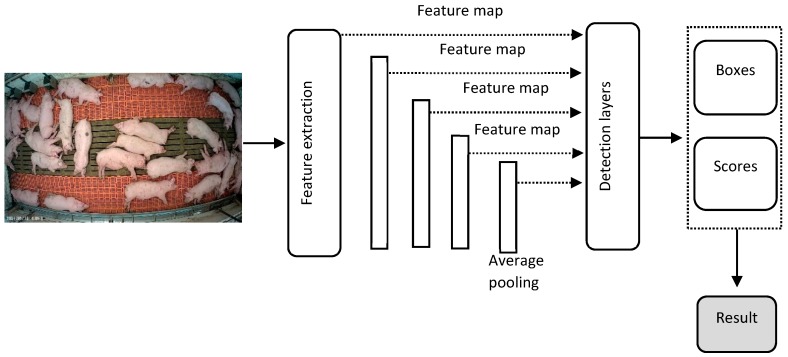
Schematic diagram of the single shot multibox detector (SSD) used in this study.

The SSD was proposed by Liu et al. [30] and is based on a bounding box regression principle. In the SSD, the input images are first divided into small kernels on different feature maps to predict anchor boxes of each kernel (Figure 5). In comparison with the Faster R-CNN and R-FCN techniques, SSD uses a single feed forward CNN to produce a series of bounding boxes and scores for the presence of the objects in each box [29]. Finally, the classification scores of each region are computed based on the score obtained in the previous section. The SSD model, instead of applying proposal generation, encloses the whole process into a single network, which leads to less computational time.

The initial learning rate is a hyperparameter and shows adjustment of weight of the networks. 

Too high learning rates may cause poor converge or a network unable to converge, and a low initial learning rate will result in slow convergence [31]. So, in this study different initial learning rates of 0.03, 0.003, 0.0003 were selected to train the Faster R-CNN, R-FCN and SSD networks. The momentum algorithm accumulates the weighted average of the previous training gradient and continues to move in that direction. The past weighted average updates the current gradient direction, and the momentum coefficient specifies to what extent the update needs to increase or decrease [32]. In this study, training of all networks was conducted with a momentum 0.9. The right number of iterations is important in training of the models, as too small numbers will result in poor performance while a high number of iterations may cause a weakening of the generalization ability of the trained model [32]. So, according to the number of samples [32] the iteration number, which shows the number of weight updates during the training process, was chosen to be 70,000 for all networks in this study. Furthermore, for data augmentation a random horizontal flip method for all detectors was used. 

## 3. Experimental Results and Discussion 

The big challenges in monitoring of pigs’ behaviour, particularly in large-scale farms, are cost, time and labour-intensity of the visual investigation. The importance of automatic monitoring of group and individual behaviours of pigs has led to the development of image processing techniques with the ability to monitor a large number of pigs. However, due to the variability in farm and animal conditions, especially in commercial farms, the development of a robust monitoring technique with the ability to detect individual animal behaviours has been examined in this study. 

The performance obtained for each posture class of the models in the test data set with each initial learning rate is illustrated in Table 2. In order to evaluate the detection capability of the proposed models, the widely used average precision (AP) of each class and mean average precision (mAP) were calculated. High values for the AP and mAP show the acceptable performance of the R-FCN ResNet101, Faster R-CNN ResNet 101 and Faster R-CNN Inception V2 detection approaches for scoring of standing, lying on belly and lying on side postures among group-housed pigs when compared to the other models. Additionally, the learning rate of 0.003 gave the best results in the mentioned detection models. The evaluation metrics illustrate a trend for improvement when the learning rate decreases from 0.03 to 0.003, however these values declined at the learning rate of 0.0003. This finding is in line with the previous finding that the detection performance changes with the same trend in learning rates in various scenarios [33]. 

**Figure 6:**
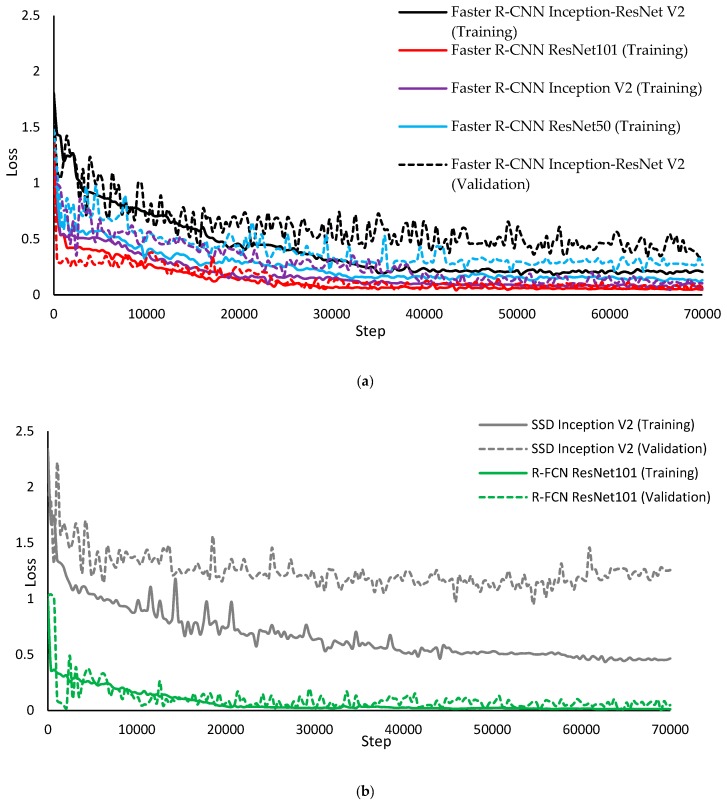
Training and validation loss during the (**a**) Faster R-CNN, (**b**) R-FCN and SSD training processes.

The training and validation loss of the models at a learning rate of 0.003 are presented in Figure 6. As can be seen, there are more fluctuations in the validation curves than in the training ones for each model, which could be due to the lower size of data set used in the validation phase [34]. The graphs show the decrease in the loss of training and validation steps of the developed models, and the values converged at a low value when the training process was finished, which is an indicator of a sufficiently trained process. After training the model, 400 images (not used for training and validation) were used as a test set (including 4987 lying and standing postures) to evaluate the correctness of the posture detection approaches. The test data set contained images from the three farms of the study which were selected from different times of the day where animals had lying, feeding, drinking and locomotion activities in the barn. 

**Figure 7:**
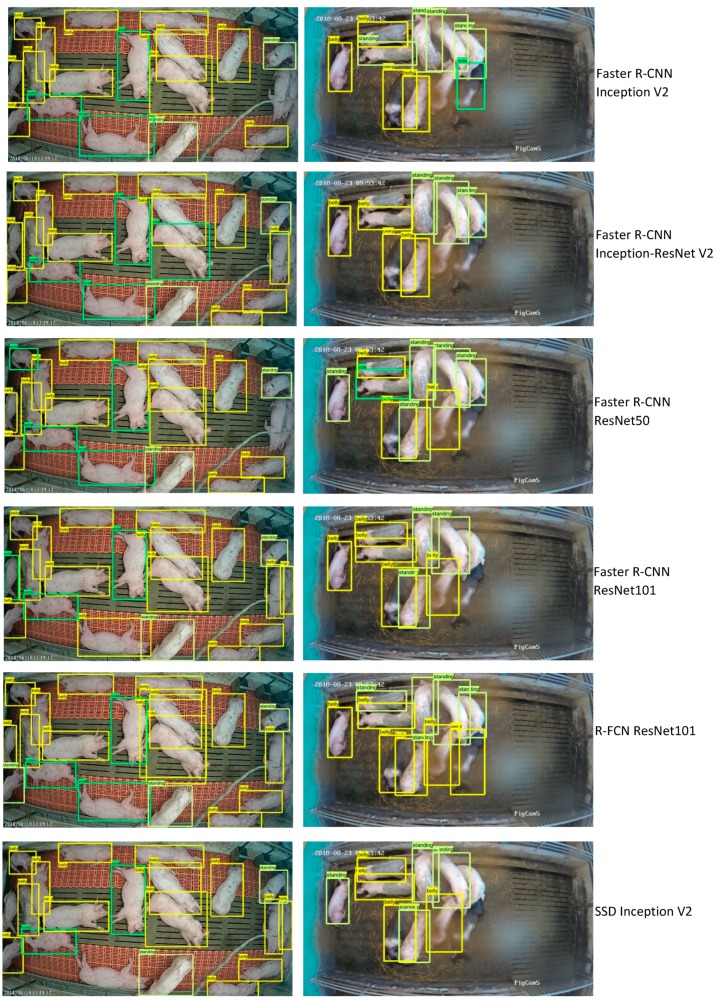
Examples of detected standing (light green rectangle), lying on belly (yellow rectangle) and side posture (green rectangle) of six different models in various farming conditions.

Figure 7 illustrates examples of detected postures by Faster R-CNN, R-FCN and SSD models in the different farming conditions in the learning rate of 0.003. As can be seen from the figure, some of the developed algorithms have the ability to detect different lying and standing postures under all tested commercial farming conditions. In some cases, there were multiple postures in an image, so the possibility of detecting various postures in an image is also shown in Figure 7. 

**Figure 8:**
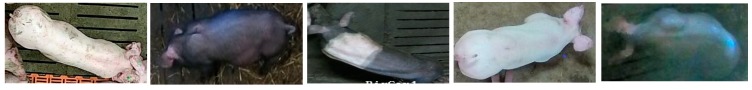
Sample of images of standing posture which are similar to belly lying posture in top view.

It can be observed from Table 2 that one of the major misdetections was with respect to standing and belly lying postures. Due to the similarity between these postures in top view images (Figure 8), some incorrect detection was observed. However, in lying on side the animals have a different top view image (Figure 2) compared to the other postures, and the number of incorrect detections was lower. 

The confusion matrix of the selected R-FCN ResNet101 model in the test dataset is shown in Table 3. As can be seen in the table, the number of misdetections between standing and belly lying postures are higher than the other postures. 

The proposed R-FCN ResNet101 technique for detection of standing and lying postures in pigs under commercial farming conditions by 2D cameras provided a high level of detection performance (e.g., mAP of 0.93), which is illustrated in Figure 7. In a sow posture study, Faster R-CNN along with RPN and ZF-net models were able to detect standing, sitting and lying behaviours with a mAP of 0.87 using Kinect v2 depth images [16]. In another study, monitoring of lying and nursing behaviour of sows using a deep learning approach (FCN and VGG16) resulted in achieved accuracy, sensitivity and specificity of 96.4%, 92.0% and 98.5%, respectively [17]. However, the study reported by [17] was carried out to monitor one sow per image, which has less probability of detection mistake compared to our study with number of pigs varying from 7–32 per image. The use of Faster R-CNN as a detector and ZF-net as a feature extractor showed high values of precision and recall (99.6% and 86.93%, respectively) in a study by [18]. However, compared to our study, they had just four animals in each image and each animal’s body was labelled as A, B, C, and D for tracking their behaviours, which would help to improve detection performance. There were some misdetections of the test data set for each posture in our study. This was mainly due to the similarity between standing and belly lying postures in top view images (Figure 8, as previously discussed) and to the problem of covering of the camera lens with fly dirt as time progressed, reducing the visibility in images [10]. Furthermore, the shape change of an animal’s body caused by the fisheye lens effect at the edge of the pen (particularly in the weaning farm), and the existence of a water pipe in the middle of the pen (at the fattening farm) which caused some invisible area in the image, gave some misdetection in the models. Low resolution of the image data impacts on the performance of machine vision techniques, and the model is not able to extract enough features to perform accurate detection. As shown in this study, misdetection was more likely for pigs with black skin colour as they have a greater similarity with the background (pen floor) colour. 

Furthermore, in this study testing time of the state-of-the-art detection models was calculated. The speed of each proposed method was measured based on the frames per second (FPS) [35]. The results show a FPS of 6.4 for the Faster R-CNN Inception V2, 3.6 for the Faster R-CNN ResNet50, 3.8 for the Faster R-CNN ResNet101, 2.3 for the Faster R-CNN Inception-ResNet V2, 5 for the R-FCN ResNet101, and 8.8 for the proposed SSD Inception V2. According to Table 2, R-FCN ResNet101 showed the highest performance as well as an acceptable FPS (5) for the pig posture detection. It was also found that the Faster R-CNN Inception V2 had high enough performance for lying-on-side posture detection along with a FPS of 6.4. Since the lying posture can be used as a parameter of pig comfort assessment in various thermal conditions, the Faster R-CNN Inception V2 model can be beneficial in terms of higher processing speed. The SSD model had the highest rate of FPS (8.8), however the lowest performance was obtained in this model, which was not sufficiently reliable in pig lying and standing detection. 

**Figure 9:**
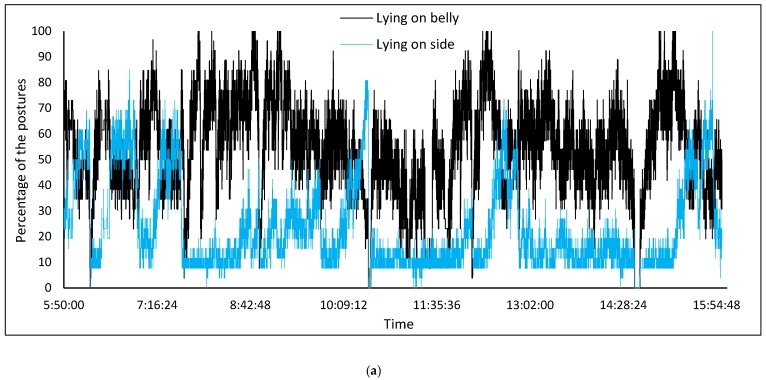

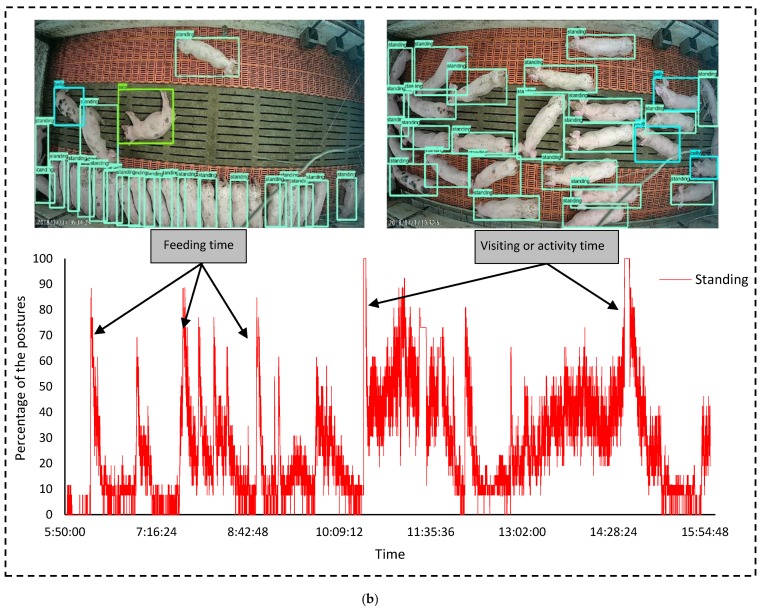
(**a**) Results of scoring (in percentage) of the lying and standing postures across the day. (**b**) Standing posture (light blue rectangle), lying on belly (blue rectangle) and side posture (green rectangle).

The proposed R-FCN model could be used to monitor pig postures in the group to control climate and barn conditions continuously. In this context, three days of recorded video data were fed into the model in order to score the number of the lying and standing postures. Results of the scoring phase are shown in Figure 9. Data from early morning (5:50) until evening (16:00), which includes different animals’ activities, were utilised and the number of the postures continuously saved in excel files. 

As shown in the scoring diagrams, the algorithm can continuously monitor the activity and postures of group-housed pigs in the barn. In the graph of standing postures, activity peaks were apparent at feeding and activity times or when the animals were checked by the farmer. The lying and standing activity patterns of the animals, which were scored using the proposed automated method, offer the advantage of early detection of health and welfare issues in pig farms [3]. For example, changes in lying and standing activities levels could be used for the detection of lameness and tail biting events [36].

In a previous study, Nasirahmadi et al. [12] showed that image processing and a linear SVM model was able to score different lying postures (sternal and lateral) in commercial farming conditions. However, the performance of the scoring technique was highly dependent on the output of the image processing method, which led to some wrong scoring in the pigs lying postures. In this study, due to the use of different deep learning approaches, high enough precision for practical use was obtained. The monitoring approach described in this study could be a valuable tool to detect changes in the number of pigs in the standing, lying on side or belly postures to improve welfare and health surveillance. However, this new method needs to be adapted and evaluated with a wider range of farming conditions (both commercial and research) in future, which may require a greater number of images for training of the model or use of other feature extraction methods. 

## 4. Conclusions

In pig husbandry, it is important to monitor the animals’ behaviour continuously between birth and slaughter. However, this can only be achieved using robust machine learning and machine vision techniques capable of processing image data from varied farming conditions. In this study, two commercial farms (weaning and fattening) in Germany and one commercial fattening farm in Sweden were used to record 2D video data for a year and provide an image database. In order to have various imaging conditions, pens with different floor type and colour, various numbers of pigs per pen (from 7 to 32) and varying ages and colours of pig were selected. The techniques proposed in this study were based on using image data from the surveillance system and Faster R-CNN, R-FCN and SSD methods. These models were trained using 80% (3600) of the image data and validated by 20% (900) of the 2D images, with a total of around 52,785 standing, lying on belly and lying on side postures in the images. The trained model was then evaluated using 400 new images. Results of the testing phase showed a high level of mAP and good processing speed for the different postures in the R-FCN ResNet101 model. This technique performed well in the monitoring of various lying and standing postures of pigs in image data acquired from commercial farms, which has been the limitation of most 2D and 3D conventional machine vision techniques. The proposed model has the flexibility and good robustness toward different pig farm lighting conditions, age of the animals and skin colour, which can be an important step toward developing real-time, continuous computer-based monitoring of livestock farms. 

## Figures and Tables

**Table 1:** Details of image data sets used for posture detection.

Posture Classes	Training Process			Testing Process
	Number of Individual Postures			
	Training	Validation	Total	Test
Standing	11,632	4372	16,004	1839
Lying on side	11,435	4085	15,520	1489
Lying on belly	15,781	5480	21,261	1659
Total samples	**38,848**	**13,937**	**52,785**	**4987**

**Table 2:** Performance of the detection phase in test data set in various learning rates.

			AP			
Classes	Learning Rate	Feature Extractor	Standing	Lying on Side	Lying on Belly	mAP
**Faster R-CNN**	0.03	Inception V2	0.82	0.87	0.88	0.86
**Faster R-CNN**	0.03	ResNet50	0.80	0.85	0.83	0.83
**Faster R-CNN**	0.03	ResNet101	0.87	0.86	0.81	0.85
**Faster R-CNN**	0.03	Inception-ResNet V2	0.79	0.83	0.77	0.80
**R-FCN**	0.03	ResNet101	0.88	0.88	0.87	0.88
**SSD**	0.03	Inception V2	0.69	0.70	0.68	0.69
**Faster R-CNN**	0.003	Inception V2	0.90	0.93	0.91	0.91
**Faster R-CNN**	0.003	ResNet50	0.85	0.92	0.89	0.88
**Faster R-CNN**	0.003	ResNet101	0.93	0.92	0.89	0.91
**Faster R-CNN**	0.003	Inception-ResNet V2	0.86	0.89	0.84	0.86
**R-FCN**	0.003	ResNet101	0.93	0.95	0.92	0.93
**SSD**	0.003	Inception V2	0.76	0.79	0.74	0.76
**Faster R-CNN**	0.0003	Inception V2	0.85	0.90	0.89	0.87
**Faster R-CNN**	0.0003	ResNet50	0.85	0.86	0.87	0.86
**Faster R-CNN**	0.0003	ResNet101	0.87	0.89	0.87	0.88
**Faster R-CNN**	0.0003	Inception-ResNet V2	0.80	0.85	0.79	0.81
**R-FCN**	0.0003	ResNet101	0.90	0.90	0.88	0.89
**SSD**	0.0003	Inception V2	0.75	0.80	0.72	0.76

**Table 3:** Confusion matrix of the proposed R-FCN ResNet101 in the test data set at learning rate of 0.003.

		Predicted Class		
**Actual class**		**Standing**	**Lying on side**	**Lying on belly**
	Standing	1672	25	89
	Lying on side	18	1382	31
	Lying on belly	71	16	1523
